# Clinical and functional characteristics of individuals with alpha-1 antitrypsin deficiency: EARCO international registry

**DOI:** 10.1186/s12931-022-02275-4

**Published:** 2022-12-16

**Authors:** Marc Miravitlles, Alice M. Turner, María Torres-Duran, Hanan Tanash, Carlota Rodríguez-García, José Luis López-Campos, Jan Chlumsky, Catarina Guimaraes, Juan Luis Rodríguez-Hermosa, Angelo Corsico, Cristina Martinez-González, José María Hernández-Pérez, Ana Bustamante, David G. Parr, Francisco Casas-Maldonado, Ana Hecimovic, Wim Janssens, Beatriz Lara, Miriam Barrecheguren, Cruz González, Jan Stolk, Cristina Esquinas, Christian F. Clarenbach

**Affiliations:** 1 Pneumology Department, Hospital Universitari Vall d’Hebron; Vall d’Hebron Institut de Recerca (VHIR), Vall d’Hebron Barcelona Hospital Campus, Passeig Vall d’Hebron 119-129, 08035 Barcelona, Spain; 2 Centro de Investigación Biomédica en Red de Enfermedades Respiratorias (CIBERES), Instituto de Salud Carlos III, Madrid, Spain; 3 Respiratory Medicine, University Hospitals Birmingham NHS Foundation Trust, Birmingham, UK; 4 Institute of Applied Health Research, University of Birmingham, Birmingham, UK; 5 Servicio de Neumología. Hospital Álvaro Cunqueiro. NeumoVigo I+I Research Group, IIS Galicia Sur, Vigo, Spain; 6 Department of Respiratory Medicine and Allergology, Skåne University Hospital, Lund University, Malmö, Sweden; 7 Servicio de Neumología, Complejo Hospitalario Clínico-Universitario de Santiago, Santiago de Compostela, Spain; 8 Unidad Médico-Quirúrgica de Enfermedades Respiratorias. Instituto de Biomedicina de Sevilla (IBiS), Hospital Universitario Virgen del Rocío/Universidad de Sevilla, Sevilla, Spain; 9 Department of Pneumology, Thomayer Hospital, First Faculty of Medicine, Charles University, Prague, Czech Republic; 10 Pulmonology Department, Hospital da Senhora da Oliveira, Guimarães, Portugal; 11 Servicio de Neumología. Hospital Clínico de San Carlos. Departamento de Medicina, Facultad de Medicina, , Universidad Complutense de Madrid, Madrid, Spain; 12 Research Institute of Hospital Clínico San Carlos (IdISSC), Madrid, Spain; 13 Pneumology Unit, IRCCS San Matteo Hospital Foundation, Pavia, Italy; 14 Department of Internal Medicine and Therapeutics, University of Pavia, Pavia, Italy; 15 Pneumology Department, Hospital Universitario Central de Asturias, Instituto de Investigacion Sanitaria del Principado de Asturias, Oviedo, Spain; 16 Pneumology Department, Hospital Universitario Nuestra Señora de La Candelaria, Santa Cruz de Tenerife, Spain; 17 Pneumology Section, Hospital Sierrallana-TresMares , Cantabria, Spain; 18 Department of Respiratory Medicine, University Hospitals of Coventry and Warwickshire, Clifford Bridge Road, Coventry, UK; 19 Servicio de Neumología. Hospital Clínico Universitario San Cecilio. Departamento de Medicina, Facultad de Medicina, Universidad de Granada, Granada, Spain; 20 Clinic for Respiratory Diseases, University Hospital Center Zagreb, Zagreb, Croatia; 21 School of Medicine, University of Zagreb, Zagreb, Croatia; 22 Laboratory of Respiratory Diseases, Department of Chronic Disease, Metabolism and Ageing, Katholieke Universiteit (KU) Leuven, Leuven, Belgium; 23 Department of Respiratory Diseases, University Hospitals Leuven, Leuven, Belgium; 24 Servicio de Neumología, Hospital Clínico Universitario de Valencia. Instituto de Investigación INCLIVA, Valencia, Spain; 25 Department of Pulmonology, Leiden University Medical Center, Leiden, The Netherlands; 26 Division of Pulmonology, University Hospital Zurich, Zurich, Switzerland

**Keywords:** Registry, Alpha-1 antitrypsin, Phenotypes

## Abstract

**Background:**

Alpha-1 antitrypsin deficiency (AATD) is a rare disease that is associated with an increased risk of pulmonary emphysema. The European AATD Research Collaboration (EARCO) international registry was founded with the objective of characterising the individuals with AATD and investigating their natural history.

**Methods:**

The EARCO registry is an international, observational and prospective study of individuals with AATD, defined as AAT serum levels < 11 μM and/or proteinase inhibitor genotypes PI*ZZ, PI*SZ and compound heterozygotes or homozygotes of other rare deficient variants. We describe the characteristics of the individuals included from February 2020 to May 2022.

**Results:**

A total of 1044 individuals from 15 countries were analysed. The most frequent genotype was PI*ZZ (60.2%), followed by PI*SZ (29.2%). Among PI*ZZ patients, emphysema was the most frequent lung disease (57.2%) followed by COPD (57.2%) and bronchiectasis (22%). Up to 76.4% had concordant values of FEV1(%) and KCO(%). Those with impairment in FEV1(%) alone had more frequently bronchiectasis and asthma and those with impairment in KCO(%) alone had more frequent emphysema and liver disease. Multivariate analysis showed that advanced age, male sex, exacerbations, increased blood platelets and neutrophils, augmentation and lower AAT serum levels were associated with worse FEV1(%).

**Conclusions:**

EARCO has recruited > 1000 individuals with AATD from 15 countries in its first 2 years. Baseline cross sectional data provide relevant information about the clinical phenotypes of the disease, the patterns of functional impairment and factors associated with poor lung function.

*Trial registration* www.clinicaltrials.gov (ID: NCT04180319)

**Supplementary Information:**

The online version contains supplementary material available at 10.1186/s12931-022-02275-4.

## Introduction

Alpha-1 antitrypsin deficiency (AATD) is a rare disease that is associated with an increased risk of pulmonary emphysema in adults, and liver disease and panniculitis in adults and children [1]. It has been estimated that approximately 1/3500 and 1/6000 individuals of European descent may be affected by severe AATD in its homozygous PI*ZZ form [2] and that around 1/800 patients with chronic obstructive pulmonary disease (COPD) in Europe have severe AATD [3].

Understanding the clinical characteristics and the natural history of a rare disease can be challenging due to the lack of large cohorts. This is even more challenging in the case of AATD because of the important influence of external factors, such as smoking, alcohol consumption or other toxic exposures, on the clinical manifestations in patients with the disease [1, 4]. Traditionally, some European countries have organised national registries that have had different success in collecting prospective follow-up data of patients with AATD [5–7]. Some 20 years ago, the Alpha-1 International Registry (AIR) tried to harmonise the collection of prospective data from different European and non-European countries and included more than 4000 patients with severe AATD [8], but less than 400 had long-term follow-up [9]. The standardised collection of follow-up data of patients with AATD from different countries and under different regimens of treatment in a large international registry was an unmet need identified by patients and researchers [10]. Similarly, the European Commission [11] and the European Respiratory Society (ERS) [12] recommended the setup of large international registries to collect structured, prospective data to better understand the natural history of AATD.

The European Alpha-1 antitrypsin Deficiency Research Collaboration (EARCO) international registry is an initiative of the EARCO Clinical Research Collaboration (CRC) of the ERS, with the objective of characterising AATD of different genotypes and investigating their natural history and the impact of different treatments, including augmentation therapy [13, 14]. Although the EARCO registry was created as a European initiative, it has extended beyond European boundaries to become a global registry. In this article we describe the characteristics of individuals with AATD included in the registry during the first 2 years, compare the characteristics of augmented versus non augmented patients and the factors associated with impaired lung function in individuals with the PI*ZZ genotype.

## Method

### Structure of EARCO

The EARCO international registry is an observational, multicenter, international study to describe the natural history of subjects with AATD [14]. Participation in the registry is open to every clinician who manages patients with AATD. The observational design implies that patients are treated and followed according to the attending physicians’ criteria. The EARCO steering committee comprises pulmonologists, researchers and patients’ representatives, headed by two co-chairs.

The study protocol received central ethics approval by the research ethics committee of the Vall d’Hebron University Hospital of Barcelona, Spain (PR(AG)480/2018) and was subsequently approved by all participating centres. All participants provided written informed consent. The EARCO registry protocol has been registered in www.clinicaltrials.gov (ID: NCT04180319), published [14] and is hosted in www.earco.org. The personal data of the patients are kept under strict confidentiality in compliance with the provisions of the General Data Protection Regulation (GDPR) 2016/679 of the European Parliament and of the European Council of April 27th, 2016.

In countries with more than one recruiting center, there is a national coordinator that has access to fully anonymized data of the patients from their country for analysis and evaluation. Despite being an international registry, there is the possibility to build national registries in EARCO with an independent management [15–17].

### Objectives of the EARCO registry

The main objectives of the EARCO registry are: (1) to generate longitudinal long-term, high-quality clinical data of individuals with AATD; (2) to understand the natural history and prognosis of AATD; (3) to investigate the effect of AAT augmentation and other therapies on the progression of lung disease, and (4) to learn more about the course of the disease in patients with severe AATD with genotypes other than PI*ZZ.

In the current publication, we describe the characteristics of the individuals included in the EARCO registry from its launch in February 2020 to May 2022.

### Population and measurements

The inclusion criteria are: (1) individuals with diagnosed AATD; (2) deficiency is defined as AAT serum levels < 11 μM (50 mg/dL) and/or proteinase inhibitor genotypes PI*ZZ, PI*SZ and compound heterozygotes or homozygotes of other rare deficient variants. The only exclusion criteria are having at least one normal M allele or lack of patient consent.

The data collected include: demographics, proteinase inhibitor genotype, comorbidities, lung function, respiratory symptoms, ultrasound-based elastography of the liver, exacerbations of respiratory disease, quality of life measured by the COPD Assessment Test (CAT) specific questionnaire [18] and the EuroQoL (EQ) 5D-3L generic questionnaire [19], physical activity measured by the Physical Activity Vital Sign (PAVS) [20] and the mean time walked per day [21], chest computed tomography (as applicable) and treatment.

Data are entered into a secure database through an electronic case report form (eCRF) hosted in the EARCO website (www.earco.org). Data are centrally monitored, and queries are sent for missing or invalid data.

### Statistical analysis

Comparison of characteristics between augmented and non-augmented PI*ZZ subjects were conducted by the Student’s t-test or Mann–Whitney U-test (if normality was not assumed) in case of quantitative variables. The Chi-squared test (Fisher test for frequencies < 5) was used for the comparison of categorical variables.

The Fibrosis-4 (FIB-4) score was calculated as age (years) × AST [IU/L]/(platelet count [10^9^/L]  × √ALT [IU/L]). A FIB-4 value < 1.45 has a high negative predictive value for ruling out advanced fibrosis and > 3.25 a high specificity and a 65% positive predictive value for ruling in advanced fibrosis.

Impairment in FEV1(%), KCO(%) or both was considered when values were < 80% of reference; the Kappa index was carried out to analyse the concordance between them. Comparison of characteristics of PI*ZZ patients according to their type of impairment in lung function was performed by the Anova test with Bonferroni correction for multiple comparisons. Linear relationships were analysed using Pearson’s correlation coefficient.

Linear regression models for all PI*ZZ, augmented and non-augmented PI*ZZ subjects were performed to identify variables related with FEV1(%). Clinical variables of interest were included as independent factors. The results were described with beta coefficients (B), 95% confidence interval (CI) and p-values. For all the tests, p-values < 0.05 were considered statistically significant. The statistical package R Studio (V2.5.1) was used for the analyses.

## Results

### Participating centers and characteristics of the population

The EARCO international registry was launched in February 2020 and by May 2022 there were 47 recruiting centers in 15 countries. The database included 1079 individuals, of which 35 (3.2%) were excluded, 20 because there was no information about AAT genotype and 15 had a normal M allele, leaving 1044 subjects for analysis.

**Fig. 1:**
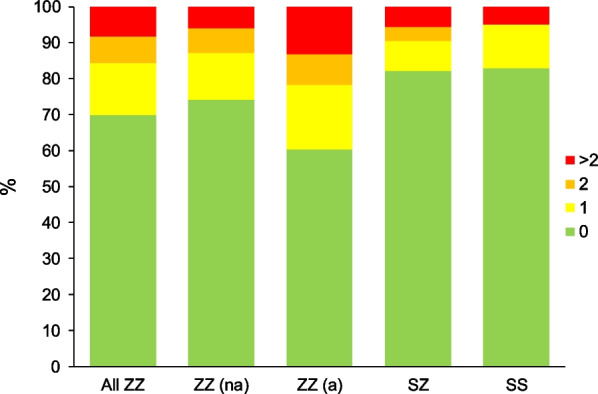
Frequency of exacerbations of lung disease in participants in the EARCO registry according to genotype. ZZ(na): non-augmented PI*ZZ; ZZ(a): Augmented PI*ZZ

The most frequent genotype was PI*ZZ (629, 60.2%), followed by PI*SZ (305, 29.2%), PI*SS (41, 3.9%) and rare variants (69, 6.6%) (Additional file 1: Table S1). Regarding the Pi*ZZ participants, their mean age was 55.6 years (standard deviation (SD): 13.2), the age at diagnosis was 44.7 years (SD: 16.7), and 51.5% were male. Only 1.8% were active workers and 72.8% were index cases (i.e. diagnosed due to their presentation with symptoms consistent with AATD), with lung disease reported by 81.4%. Emphysema was the most frequent lung disease (57.2%) followed by COPD (55.9%) and bronchiectasis (22%), only 5 patients had panniculitis. The majority of Pi*ZZ participants were non exacerbators (Fig. 1), and 29.3% had a history of pneumonia. Comorbidities were frequent, with an age corrected Charlson index of 3.3 (SD: 1.9) and 26.1% had a cardiovascular disease (Table 1).

**Table 1:** Demographic and clinical characteristics of patients included in the EARCO registry and comparison between augmented and non-augmented PI*ZZ patients

Variables	All ZZ (n = 629)	ZZ non-augmented (n = 436)	ZZ augmented (n = 190)	p value
Age, years	55.6 (13.2)	54.4 (14.0)	58.4 (10.9)	0.009
Sex, male (%)	324 (51.5)	208 (47.7)	114 (60)	0.005
BMI (kg/m^2^)	26 (4.8)	26 (4.8)	26.1 (4.8)	0.629
Smokers (%)	11 (1.8)	10 (2.3)	1 (0.5)	0.187
Ex-smokers (%)	356 (56.8)	202 (46.3)	152 (80.9)	< 0.001
Never smokers (%)	260 (41.5)	224 (51.4)	35 (18.6)	< 0.001
Tobacco Exposure (Pack-years)	17.8 (14)	14.2 (12.2)	22.7 (14.8)	< 0.001
Working status				
Active	338 (53.8)	263 (60.5)	72 (37.9)	< 0.001
Retired	235 (37.4)	135 (31)	100 (52.6)	< 0.001
Unemployed	30 (4.8)	15 (3.4)	15 (7.9)	0.029
Other	25 (4.0)	22 (5.1)	3 (1.6)	0.069
Age at diagnosis of AATD	44.7 (16.7)	43.1 (17.9)	48.3 (13.0)	0.010
Reason for diagnosis				
Index case (%)	457 (72.8)	302 (69.4)	152 (80.0)	0.006
Family screening (%)	115 (18.3)	100 (22.9)	15 (7.9)	< 0.001
Clinical characteristics				
Chronic respiratory disease (%)	508 (81.4)	317 (73.5)	188 (98.9)	< 0.001
COPD (%)	349 (55.9)	180 (41.8)	167 (87.9)	< 0.001
Emphysema (%)	357 (57.2)	187 (43.4)	169 (88.9)	< 0.001
Bronchiectasis (%)	137 (22)	87 (20.2)	49 (25.8)	0.120
Asthma (%)	88 (14.1)	74 (17.2)	14 (7.4)	0.001
Liver disease (%)	99 (16)	69 (16.1)	30 (16)	0.981
Charlson index (*age corrected*)	3.3 (1.9)	3 (1.9)	4 (1.8)	< 0.001
Cardiovascular disease (%)	163 (26.1)	91 (20.9)	72 (38.5)	< 0.001
Exacerbations previous year (%)	189 (30.2)	113 (25.9)	75 (39.7)	< 0.001
History of pneumonia (%)	173 (29.3)	113 (28)	57 (30.8)	0.491

Data are presented a n (% of patients with non-missing data) or mean (Standard deviation)

BMI: Body mass index; COPD: Chronic obstructive pulmonary disease; AATD: Alpha-1 antitrypsin deficiency; cardiovascular disease includes hypertension, ischaemic heart disease, congestive heart failure and peripheral vascular disease

**Fig. 2:**
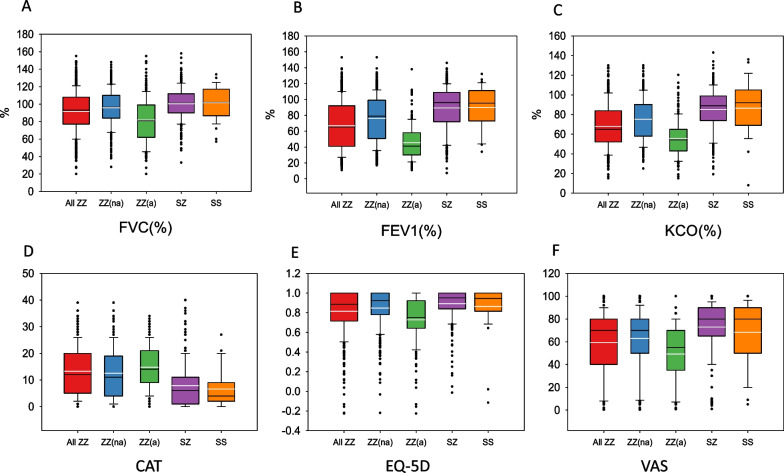
Distribution of values of lung function and quality of life according to the different genotypes. ZZ(na): non-augmented PI*ZZ; ZZ(a): Augmented PI*ZZ. In each box plot, the median value is indicated by the center horizontal black line, the 25th and 75th percentiles are indicated by the lower and upper box horizontal lines, and the mean value is indicated by the center horizontal white line. Whiskers above and below the box indicate the 90th and 10th percentiles. Circles on the high end indicate the outliers

The mean FEV1(%) of Pi*ZZ participants was 66.9% (SD: 30.7%) and mean KCO(%) 68% (SD: 23.2%). In general participants showed a moderate impairment in quality of life with a mean CAT score of 13.2 (SD: 9.3) and EQ-5D 0.82 (SD: 0.22) and VAS 59.3 (SD: 28.2). The remaining spirometric, quality of life and activity variables, as well as the results of the blood analysis are summarised in Table 2. Differences in LFTs and quality of life parameters between different genotypes are shown in Fig. 2.

**Table 2:** Lung function, blood analysis, health related quality of life and physical activity of patients with PI*ZZ genotype and comparison between augmented and non-augmented subjects

Variables	All ZZ (n = 629)	ZZ non-augmented (n = 436)	ZZ augmented (n = 190)	p value
Lung function				
FVC, %	91.8 (23.7)	96.1 (21.3)	81.8 (26.1)	< 0.001
FEV1, %	66.9 (30.7)	76.3 (29.4)	45.5 (21.7)	< 0.001
FEV1/FVC, %	0.54 (0.2)	0.59 (0.2)	0.44 (0.1)	< 0.001
KCO, %	68 (23.2)	75.1 (22.4)	55.8 (19.1)	< 0.001
Quality of life				
CAT	13.2 (9.3)	12.5 (9.7)	14.8 (8.4)	0.001
BODEx	2 (2.0)	1.4 (1.8)	3.3 (2.0)	< 0.001
EQ-5D	0.82 (0.22)	0.85 (0.20)	0.73 (0.25)	< 0.001
VAS	59.3 (28.2)	62.9 (28.2)	49.3 (25.7)	< 0.001
Exercise/activity				
Active days in last week	2.7 (2.4)	2.7 (2.3)	2.6 (2.4)	0.717
Active days usual week	2.8 (2.4)	2.9 (2.4)	2.7 (2.4)	0.440
Minutes walked per day	58.8 (63.9)	59 (64.2)	57.7 (63.0)	0.924
Blood analysis				
Haemoglobin	14.8 (2.4)	14.5 (2.6)	15.3 (1.7)	0.001
White blood cells	7.0 (2.2)	6.7 (2.0)	7.6 (2.4)	< 0.001
Neutrophils	4.5 (3.2)	4.2 (2.5)	5.1 (4.2)	0.002
Eosinophils	0.25 (0.60)	0.22 (0.41)	0.30 (0.85)	0.121
Platelets	237.8 (73.4)	238.7 (70.9)	237.0 (77.5)	0.571
AAT (mg/dL)*	23.8 (8.3)	23.8 (8.7)	23.8 (7.5)	0.571
AST (UI/L)	29.9 (17.4)	30.8 (16.7)	27.8 (13.1)	0.202
ALT (UI/L)	29.7 (17.9)	30.1 (17.5)	28.7 (19.0)	0.474
GGT (UI/L)	43.6 (44.1)	41.5 (45.6)	47.8 (40.8)	0.001
FIB-4	1.6 (2.5)	1.6 (2.1)	1.8 (3.3)	0.103

Values are mean (Standard deviation) unless otherwise specified

FVC: Forced vital capacity; FEV1: Forced expiratory volume in 1 s; KCO: Carbon monoxide transfer coefficient; CAT: COPD assessment test; BODEx: Body mass index, obstruction, dyspnoea, exacerbations index; EQ-5D: Euro QoL 5 dimensions questionnaire; VAS: Visual analogue scale; AST: Aspartate aminotransferase; ALT: Alanine aminotransferase; GGT: Gamma-glutamyl transferase; FIB-4: Fibrosis 4

^*^AAT levels were obtained at diagnosis, before initiating augmentation therapy

### Augmentation therapy: patterns of treatment and comparison of augmented versus non-augmented PI*ZZ individuals

A total of 190 (30%) PI*ZZ patients received augmentation therapy at the time of enrolment in the registry; of them 3 (1.5%) received infusions at home, 18 (9.5%) in Primary Care and the remaining 169 (88.9%) in the hospital. The treatment regimens most used were 120 mg/kg/biweekly in 93 (48.9%), 180 mg/kg/3 weekly in 55 (28.9%), 60 mg/kg/weekly in 35 (18.4%) and 2 patients (1%) at 250 mg/kg/monthly, in 5 cases the investigators indicate that they used a different regimen: 2 used 180 mg/kg/biweekly, and doses of 120 mg/kg/12 days, 140 mg/kg/biweekly and 60 mg/kg/biweekly were used by one patient each.

Augmented patients were older, more frequently male, former smokers and either unemployed or retired compared to non-augmented patients. They were more frequently index cases (79.9% versus 69.4%; p = 0.007) and with a chronic respiratory disease (98.9% versus 73.5%; p < 0.001). Only asthma was more frequent among non-augmented PI*ZZ patients (17.2% versus 7.4%; p < 0.001) (Table 1).

Augmented patients had more impaired lung function and worse quality of life, both respiratory-specific and generic; however, there were no significant differences in physical activity between augmented and non-augmented PI*ZZ individuals (Table 2). Differences in lung function and quality of life variables between augmented and non-augmented PI*ZZ are also shown in Fig. 2.

The haemoglobin levels and white blood cell counts were significantly higher in augmented PI*ZZ subjects versus non-augmented. Regarding liver function tests, only gamma-glutamyl transferase (GGT) (47.8 (SD: 40.1) IU/L vs 38.2 (SD: 45.2) IU/L; p < 0.001) was significantly higher in augmented patients (Table 2).

### Analysis of lung function of PI*ZZ individuals: concordance between FEV1(%) and KCO(%)

**Fig. 3:**
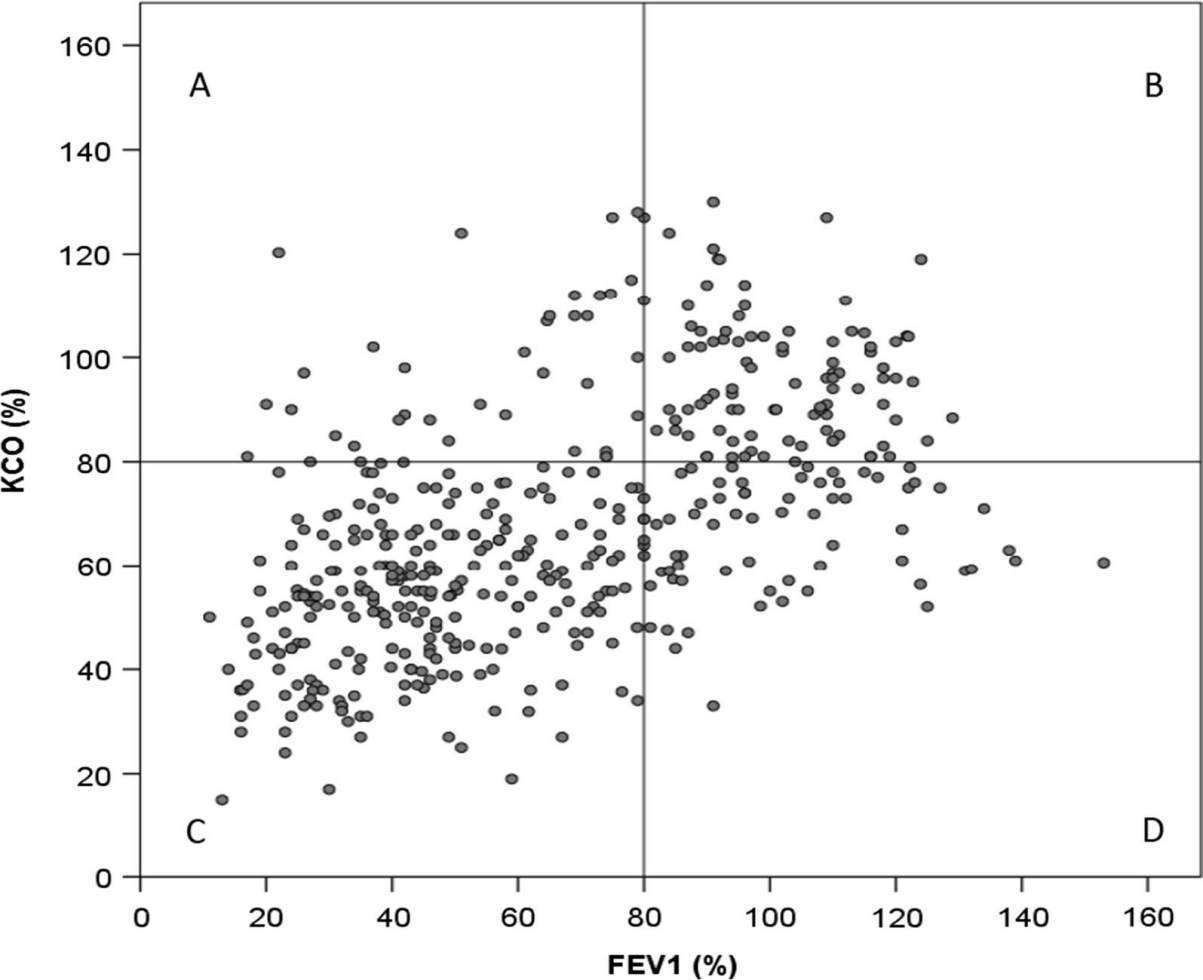
Correlation between FEV1(%) and KCO(%) in EARCO participants with PI*ZZ genotype. **A** Impaired FEV1(%) only; **B** Normal lung function tests; **C** Impaired lung function tests; **D** Impaired KCO(%) only

Of the 449 PI*ZZ participants with valid measurements of FEV1(%) and KCO(%), 343 (76.4%) had concordant values (248 (55%) both impaired and 95 (21.2%) both normal), whereas 106 (23.6%) were discordant (Fig. 3), the Kappa index was 0.47 (95% confidence interval 0.38 to 0.55).

Patients with impairment in lung function tests or FEV1(%) alone were older, more frequently male, active or former smokers and index cases, and more frequently diagnosed with a respiratory disease. Those who had impairment in FEV1(%) alone had more frequently bronchiectasis (40.5%) and asthma (24.3%). Up to 20.2% of individuals with normal lung function had asthma and 19.1% bronchiectasis. There were no significant differences in the prevalence of liver disease, although this was numerically higher in patients with impairment in KCO(%) alone (26.1%) (Table 3).

**Table 3:** Characteristics of PI*ZZ patients according to their impairment in FEV1(%), KCO(%) or both

Variables	Impaired PFTs (n = 248)	Impaired KCO(%) (n = 69)	Impaired FEV1(%) (n = 37)	Normal PFTs (n = 95)	p value
Age, years	58.7 (10.7)	55.2 (13.4)	56.2 (12.4)	45.2 (14.9)	< 0.001^cef^
Sex, male (%)	145 (58.5)	33 (47.8)	25 (67.6)	43 (45.3)	0.035
BMI (kg/m^2^)	26.1 (5.0)	25.8 (4.5)	28 (5.2)	26.1 (4.6)	0.105
Smokers (%)	4 (1.6)	2 (2.9)	1 (2.7)	2 (2.1)	0.903
Ex-smokers (%)	193 (77.8)	28 (40.6)	22 (59.5)	23 (24.2)	< 0.001^acf^
Never smokers (%)	51 (20.6)	39 (56.5)	14 (37.8)	70 (73.7)	< 0.001^acf^
Tobacco Exposure (Pack-years)	21 (14.8)	12.7 (10.8)	14 (10.2)	6.9 (7.6)	< 0.001^ac^
Working status					
Active	107 (43.1)	40 (58)	20 (54.1)	74 (77.9)	< 0.001^cef^
Retired	121 (48.8)	23 (33.3)	16 (43.2)	12 (12.6)	< 0.001^cef^
Unemployed	14 (5.6)	2 (2.9)	1 (2.7)	2 (2.1)	0.424
Other	6 (2.4)	4 (5.9)	0 (0)	7 (7.4)	0.077
Age at diagnosis of AATD	48.4 (12.8)	45.7 (18.3)	47.1 (12.7)	31.4 (22.5)	< 0.001^cef^
Reasons for diagnosis					
Index case (%)	202 (81.5)	40 (58.0)	24 (64.9)	48 (51.1)	< 0.001^ac^
Family screening (%)	24 (9.7)	19 (27.5)	6 (16.2)	30 (31.6)	< 0.001^ac^
Clinical characteristics					
Chronic respiratory disease (%)	247 (99.6)	48 (70.6)	32 (86.5)	43 (45.7)	< 0.001^abcef^
COPD (%)	214 (83.3)	19 (27.9)	25 (67.6)	5 (5.3)	< 0.001^abcdef^
Emphysema (%)	203 (81.9)	30 (44.1)	27 (73.0)	10 (10.6)	< 0.001^acdef^
Bronchiectasis (%)	45 (18.1)	23 (33.8)	15 (40.5)	18 (19.1)	0.002^ab^
Asthma (%)	27 (10.9)	8 (11.8)	9 (24.3)	19 (20.2)	0.036
Liver disease (%)	35 (14.5)	18 (26.1)	5 (13.5)	19 (20.2)	0.113
Charlson index (*age corrected*)	4 (1.9)	2.8 (1.9)	2.9 (1.3)	1.5 (1.4)	< 0.001^acef^
Cardiovascular disease (%)	80 (32.7)	13 (18.8)	10 (27)	12 (12.6)	0.001^c^
Exacerbations previous year (%)	103 (41.5)	13 (18.8)	12 (32.4)	8 (8.4)	< 0.001^acf^
History of pneumonia (%)	72 (31.2)	18 (26.9)	10 (30.3)	18 (19.6)	0.209

Impairment is considered when values are < 80% of reference

Data are presented a n (% of patients with non-missing data) or mean (Standard deviation)

LFT: Lung function tests; FEV1: Forced expiratory volume in 1 s; KCO: Carbon monoxide transfer coefficient; BMI: Body mass index; COPD: Chronic obstructive pulmonary disease; AATD: Alpha-1 antitrypsin deficiency; cardiovascular disease includes hypertension, ischaemic heart disease, congestive heart failure and peripheral vascular disease

Bonferroni ^a^p < 0.05 for comparison between the groups “Impaired PFTs and Impaired KCO(%)”. ^b^p < 0.05 for comparison between the groups “Impaired PFTs and impaired FEV1(%)”. ^c^p < 0.05 for comparison between the groups “Impaired PFTs and normal PFTs”. ^d^p < 0.05 for comparison between the groups “Impaired KCO(%) and impaired FEV1(%)”. ^e^p < 0.05 for comparison between the groups “Impaired KCO(%) and normal PFTs”. ^f^p < 0.05 for comparison between the groups “Impaired FEV1(%) and normal PFTs”

Regarding quality of life, CAT and EQ-5D scores were worse in patients with impaired lung function tests and impaired FEV1(%) compared with the other two groups. However, there were no significant differences in the patterns of physical activity, and patients with impaired FEV1(%) had a significantly longer time walking per day (77.3 min (SD: 76.4)) compared with the other groups (Table 4).

**Table 4:** Lung function, blood analysis, health related quality of life and physical activity of PI*ZZ patients according to their impairment in FEV1(%), KCO(%) or both

Variables	Impaired PFTs (n = 248)	Impaired KCO(%) (n = 69)	Impaired FEV1(%) (n = 37)	Normal PFTs (n = 95)	p value
Lung function					
FVC, %	84.7 (22.3)	111.8 (19.9)	82.6 (21.3)	103.9 (13.4)	< 0.001^acdf^
FEV1, %	44.9 (16.8)	101.2 (17.8)	54.6 (20.1)	101.8 (12.3)	< 0.001^acdf^
FEV1/FVC, %	0.42 (0.12)	0.72 (0.11)	0.51 (0.13)	0.80 (0.08)	< 0.001^abcdf^
KCO, %	53.2 (13.9)	65.5 (10.4)	97.4 (14.4)	96.7 (12.1)	< 0.001^abcde^
Quality of life					
CAT	16.7 (8.5)	8.6 (7.1)	12.9 (8.9)	5.9 (6.7)	< 0.001^acf^
BODEx	3.1 (1.9)	0.4 (0.7)	1.9 (1.9)	0.2 (0.4)	< 0.001^abcdf^
EQ-5D	0.74 (0.24)	0.9 (0.14)	0.81 (0.18)	0.93 (0.10)	< 0.001^acf^
VAS	53.6 (24.1)	73.1 (17.0)	61.6 (24.1)	76.4 (26.6)	< 0.001^acf^
Exercise/activity					
Active days in last week	2.7 (2.4)	2.7 (2.4)	2.7 (2.3)	2.6 (2.2)	0.994
Active days usual week	2.8 (2.4)	3.0 (2.3)	3.1 (2.4)	2.7 (2.1)	0.822
Minutes walked per day	56.9 (67.7)	60.2 (49.2)	77.3 (76.4)	64.6 (59.6)	0.034
Blood analysis					
Haemoglobin	15.4 (1.6)	14.7 (1.4)	15.1 (1.9)	14.1 (2.6)	< 0.001^acf^
White blood cells	7.5 (2.1)	6.2 (1.7)	6.8 (1.7)	6.1 (1.8)	< 0.001^ac^
Neutrophils	4.8 (2.5)	4.1 (4.7)	5.2 (7.3)	3.5 (1.3)	< 0.001^ac^
Eosinophils	0.23 (0.35)	0.18 (0.22)	0.27 (0.54)	0.24 (0.59)	0.175
Platelets	239.2 (75.7)	216.8 (65.0)	235.9 (56.0)	235.8 (66.8)	0.134
AAT (mg/dL)*	24.4 (7.6)	22.1 (9.2)	27.1 (10)	23.3 (7.5)	0.012^d^
AST (UI/L)	29.3 (14.3)	36.3 (34.3)	31.3 (13.8)	27.5 (13.9)	0.159
ALT (UI/L)	29.4 (18.6)	30.3 (16.7)	30.7 (15.2)	29.3 (19.5)	0.561
GGT (UI/L)	52.7 (52.1)	42.2 (49.7)	42.5 (39.3)	30.3 (25.2)	< 0.001^f^
FIB-4	1.7 (3)	2.4 (4.2)	1.4 (0.7)	1.1 (0.7)	< 0.001^ef^

Impairment is considered when values are < 80% of reference

Values are mean (Standard deviation) unless otherwise specified

FVC: Forced vital capacity; FEV1: Forced expiratory volume in 1 s; KCO: Carbon monoxide transfer coefficient; CAT: COPD assessment test; BODEx: Body mass index, obstruction, dyspnoea, exacerbations index; EQ-5D: Euro QoL 5 dimensions questionnaire; VAS: Visual analogue scale; AST: Aspartate aminotransferase; ALT: Alanine aminotransferase; GGT: Gamma-glutamyl transferase; FIB-4: Fibrosis 4

^*^AAT levels were obtained at diagnosis, before initiating augmentation therapy

Bonferroni ^a^p < 0.05 for comparison between the groups “Impaired PFTs and Impaired KCO(%)”. ^b^p < 0.05 for comparison between the groups “Impaired PFTs and impaired FEV1(%)”. ^c^p < 0.05 for comparison between the groups “Impaired PFTs and normal PFTs”. ^d^p < 0.05 for comparison between the groups “Impaired KCO(%) and impaired FEV1(%)”. ^e^p < 0.05 for comparison between the groups “Impaired KCO(%) and normal PFTs”. ^f^p < 0.05 for comparison between the groups “Impaired FEV1(%) and normal PFTs”

The analysis of blood parameters showed a significant difference in the plasma levels of AAT, with the highest levels in patients with impaired FEV1(%) and the lowest in those with impaired KCO(%) (27.1 mg/dL (SD: 10) versus 22.1 mg/dL (SD: 9.2); p < 0.05). In contrast, FIB-4 was significantly higher in patients with impaired KCO(%) compared with the remaining groups and they also had numerically lower levels of platelets (Table 4).

### Factors associated with impairment in FEV1(%) in multivariate analysis in PI*ZZ individuals

The multivariate analysis showed that advanced age, male sex, history of exacerbations, use of augmentation therapy and markers of inflammation (such as increased blood neutrophils and platelets) were associated with impaired FEV1(%). In contrast, increased serum levels of AAT were associated with better FEV1(%). When analysing these factors separately for augmented and non-augmented patients, the association of male sex and exacerbations remain for both groups, but markers of inflammation were only significant for the non-augmented patients (Table 5).

**Table 5:** Multivariate analysis of variables associated with FEV1(%) in individuals with PI*ZZ genotype in EARCO

Variables	FEV1(%)					
	All (n = 453)		Augmented (n = 157)		Non-augmented (n = 296)	
	Beta	95% CI	Beta	95% CI	Beta	95% CI
Age	− 0.366	− 0.543, − 0.189	NS	–	− 0.577	− 0.792, − 0.362
Sex (male)	− 10.414	− 15.087, − 5.741	− 13.460	− 20.283, − 6.638	− 8.004	− 14.006, − 2.003
Smokers	NS	–	NS	–	NS	–
Exacerbations	− 15.741	− 20.781, − 10.701	− 13.476	− 20.191, − 6.761	− 16.985	− 23.870, − 10.100
Pneumonia	NS	–	NS	–	NS	–
Blood eosinophils	NS	–	NS	–	NS	–
Blood neutrophils	− 0.060	− 0.092, − 0.027	NS	–	− 1.158	− 2.261, − 0.056
Serum AAT	27.077	22.949, 31.205	NS	–	NS	–
Platelets	− 0.565	− 0.859, − 0.125	NS	–	− 0.068	− 0.112, − 0.024
Augmentation	− 0.366	− 0.543, − 0.189	NS	–	NS	–

AAT: alpha-1 antitrypsin; CI: Confidence interval; FEV1: Forced expiratory volume in 1 s; NS: non-significant

## Discussion

Despite the severe disruption caused by the COVID-19 pandemic, the EARCO registry has been able to recruit more than 1000 individuals with severe AATD from 15 countries over the course of its first 2 years. The clinical characteristics of the participants are similar to other previous series, with a mean age of 55 years and an even distribution between sex, with 71.8% suffering from with pulmonary disease and 14% from liver disease. The majority of PI*ZZ patients had a concordant impairment in FEV1(%) and KCO(%); however, those with impairment in FEV1(%) alone more frequently had asthma or bronchiectasis and those with impairment in KCO(%) alone more often had emphysema and liver involvement. As expected, PI*ZZ patients on augmentation therapy were more severely affected by lung disease and were more frequently index cases compared with non-augmented PI*ZZ cases. Increased age, male sex, more frequent exacerbations, and elevated markers of inflammation, such as blood neutrophils and platelets, were associated with impaired FEV1(%), whereas higher levels of serum AAT were associated with better FEV1(%) in multivariate analysis.

The investigation about the characteristics and natural history of rare diseases, such as AATD, requires the development of registries to collect information about a large number of cases [12, 22]. National registries have been developed in Europe, United States (US) and Canada [5–7] and more than 20 years ago, the Alpha 1 International Registry was funded to harmonise data collection from several countries [8]. However, the greatest challenge has always been the collection of long-term follow up data in multicenter, international registries. At the end of the last century, the National Heart, Lung and Blood Institute (NHLBI) registry of patients with severe AATD recruited + 1000 individuals in the US and followed them for between 3.5 to 7 years to investigate the effectiveness of augmentation therapy in slowing the rate of decline of FEV1 [23]. In Europe, the Swedish, UK and German registries, among others, have generated relevant information about clinical phenotypes, comorbidities and factors influencing rate of decline of lung function [5, 6, 24–27]. However, despite these and other examples, there is a clear unmet need of large series of severe AATD patients from different countries, with different genotypes and receiving different treatments to provide new information about the clinical and functional characteristics of the disease and its natural history.

Almost two thirds of the individuals recruited have the most frequent deficient genotype PI*ZZ, but up to 8% are carriers of rare deficient variants. Nearly 30% were non index cases, the mean FEV1(%) and KCO(%) were around 75% and participants showed a moderate impairment in quality of life.

Only 30% of the severe deficient PI*ZZ individuals received augmentation therapy; this percentage is influenced by the recruitment of patients from countries in which augmentation is not reimbursed [28]. Nevertheless, augmented patients were more frequently index cases, and had more severe impairment in lung function and more frequent exacerbations compared with non-augmented. Interestingly, most augmented patients were receiving regimens other than the approved dosage of weekly infusions of 60 mg/kg. The most frequent regimen was biweekly administration of 120 mg/kg, followed by 180 mg/Kg every 3 weeks, and 5 patients were using other regimens, probably individual adjustments of dosage according to trough levels of serum AAT, a frequent practice that is not recommended by experts [12].

The usual pattern of lung disease in severe AATD is basal, bilateral, panlobular emphysema; however, different series have described other lung phenotypes, such as bronchiectasis, chronic bronchitis, asthma or even apical emphysema [25, 26]. Our population reflects this heterogeneity with 17.7% (22% of the PI*ZZ) having bronchiectasis and 15.1% (14.1% of the PI*ZZ) having asthma. The majority of our PI*ZZ patients (76%) had concordant impairment in FEV1(%) and KCO(%); interestingly, among the discordant subjects, those with impairment in FEV1(%) alone had more frequently bronchiectasis or asthma, and those with impairment in KCO(%) alone had signs of more liver involvement with a significantly higher index FIB-4, lower levels of platelets and a higher percentage of subjects with diagnosed liver disease. To the best of our knowledge, this observation of the possible link between a predominant impairment in diffusion capacity and more frequent or severe liver disease has not been previously reported and requires further investigation. Moreover, patients with impairment in KCO(%) also had significantly lower levels of serum AAT compared with patients with impairment in FEV1(%), suggesting a stronger relationship between lower levels of AAT and lung parenchymal disease instead of bronchial disease. Alternatively, patients with FEV1(%) impairment may be more severe and had higher levels of systemic inflammation which may results in elevated plasma levels of AAT. Concordance between FEV1/FVC and KCO was also observed by Ward et al. [29] in 70% of a group of 530 PI*ZZ patients from the UK. However, in their study the number of patients with impaired KCO alone was too small (only 8 subjects) and no description of clinical phenotypes were provided [29]. In a further study by the same group, a different rate of progression of impairment in airflow obstruction or in gas transfer in PI*ZZ according to impairment in FEV1 or KCO was demonstrated [6].

Increased serum levels of AAT in PI*ZZ individuals were significantly and independently associated with better FEV1(%) in multivariate analysis; this observation concurs with the results of the RAPID trial which described those patients with higher trough serum AAT levels had a reduced rate of decline of lung density, even in the placebo arm, in which the range of AAT trough levels was very limited [30].

Other independent variables associated with impaired lung function were male sex, exacerbations and some blood inflammatory markers. A faster decline in lung function in males has been already described [31, 32]; although the reason is unclear, among others, males are more often involved in “blue collar” occupations and exposed to environmental pollutants that may impact in lung function [24, 33]. The significant and strong association of exacerbations with impaired lung function may be bidirectional: patients with more severe impairment in FEV1(%) have an increased risk of exacerbations and more frequent exacerbations accelerate FEV1(%) decline. Our results agree with previous findings in longitudinal studies that confirm the impact of exacerbations in the natural history of lung disease in AATD [27, 31, 34] and stress the importance of prevention of exacerbations in patients with AATD and lung disease.

Patients with COPD have increased lung and systemic inflammation, and since AAT is an acute phase protein, they also have increased serum levels of AAT compared with healthy controls [35]. Some studies have suggested that there is a relationship between increased markers of systemic inflammation and the severity of lung and liver disease [36–38]. In our PI*ZZ patients, markers of systemic inflammation, such as increased neutrophils and platelets, were associated with more impaired FEV1(%), but this association was not observed in augmented patients. This may be an statistical effect due to a smaller sample size, but it may also be a consequence of the anti-inflammatory effect of the infused AAT that attenuates bronchial [39] and systemic inflammation [38, 40].

Our study has some limitations, the most important is the cross-sectional nature of data; however, there was a lack of information about the characteristics of large, international series of patients with severe AATD. On the other hand, it has the strength of a harmonised collection of data from many different countries with a single protocol and with close monitoring and quality control of the included data.

## Conclusions

The EARCO registry has shown novel information about the clinical and functional characteristics of a large, international registry of patients with AATD. EARCO is a prospective study that will provide relevant information about the natural history of AATD in the future and is the platform for the development of other clinical studies in the field.

## Supplementary Information

**Additional file 1: Table S1.** Description of rare variants identified in EARCO.

## Data Availability

The data that support the findings of this study are available from the EARCO group but restrictions apply to the availability of these data, which were used under license for the current study, and so are not publicly available. Data are however available from the authors upon reasonable request and with permission of the EARCO steering committee.

## Abbreviations

AAT: Alpha-1 antitrypsin
AATD: Alpha-1 antitrypsin deficiency
AIR: Alpha-1 International Registry
ALT: Alanine aminotransferase
AST: Aspartate aminotransferase
BMI: Body mass index
BODEx: Body mass index, obstruction, dyspnoea, exacerbations index
CAT: COPD assessment test
CI: Confidence interval
COPD: Chronic obstructive pulmonary disease
CRC: Clinical Research Collaboration
CRP: C-reactive protein
EARCO: European Alpha-1 antitrypsin Research Collaboration
eCRF: Electronic case report form
ERS: European Respiratory Society
EQ-5D: Euro QoL 5 dimensions questionnaire
FEV1: Forced expiratory volume in 1 s
FIB-4: Fibrosis 4
FVC: Forced vital capacity
GDPR: General Data Protection Regulation
GGT: Gamma-glutamyl transferase
IU: International units
KCO: Carbon monoxide transfer coefficient
NHLBI: National Heart, Lung and Blood Institute
NS: Non-significant
PAVS: Physical Activity Vital Sign
PI: Protease inhibitor
SD: Standard deviation
UK: United Kingdom
US: United States of America
VAS: Visual analogue scale
