# Correction: Clinical and functional characteristics of individuals with alpha-1 antitrypsin deficiency: EARCO international registry

**DOI:** 10.1186/s12931-023-02340-6

**Published:** 2023-02-18

**Authors:** Marc Miravitlles, Alice M. Turner, María Torres-Duran, Hanan Tanash, Carlota Rodríguez-García, José Luis López-Campos, Jan Chlumsky, Catarina Guimaraes, Juan Luis Rodríguez-Hermosa, Angelo Corsico, Cristina Martinez-González, José María Hernández-Pérez, Ana Bustamante, David G. Parr, Francisco Casas-Maldonado, Ana Hecimovic, Wim Janssens, Beatriz Lara, Miriam Barrecheguren, Cruz González, Jan Stolk, Cristina Esquinas, Christian F. Clarenbach

**Affiliations:** 1 Pneumology Department, Hospital Universitari Vall d’Hebron; Vall d’Hebron Institut de Recerca (VHIR), Vall d’Hebron Barcelona Hospital Campus, Passeig Vall d’Hebron 119-129, 08035 Barcelona, Spain; 2 Centro de Investigación Biomédica en Red de Enfermedades Respiratorias (CIBERES), Instituto de Salud Carlos III, Madrid, Spain; 3 Respiratory Medicine, University Hospitals Birmingham NHS Foundation Trust, Birmingham, UK; 4 Institute of Applied Health Research, University of Birmingham, Birmingham, UK; 5 Servicio de Neumología. Hospital Álvaro Cunqueiro. NeumoVigo I+I Research Group, IIS Galicia Sur, Vigo, Spain; 6 Department of Respiratory Medicine and Allergology, Skåne University Hospital, Lund University, Malmö, Sweden; 7 Servicio de Neumología, Complejo Hospitalario Clínico-Universitario de Santiago, Santiago de Compostela, Spain; 8 Unidad Médico-Quirúrgica de Enfermedades Respiratorias. Instituto de Biomedicina de Sevilla (IBiS), Hospital Universitario Virgen del Rocío/Universidad de Sevilla, Sevilla, Spain; 9 Department of Pneumology, Thomayer Hospital, First Faculty of Medicine, Charles University, Prague, Czech Republic; 10 Pulmonology Department, Hospital da Senhora da Oliveira, Guimarães, Portugal; 11 Servicio de Neumología. Hospital Clínico de San Carlos. Departamento de Medicina, Facultad de Medicina,, Universidad Complutense de Madrid, Madrid, Spain; 12 Research Institute of Hospital Clínico San Carlos (IdISSC), Madrid, Spain; 13 Pneumology Unit, IRCCS San Matteo Hospital Foundation, Pavia, Italy; 14 Department of Internal Medicine and Therapeutics, University of Pavia, Pavia, Italy; 15 Pneumology Department, Hospital Universitario Central de Asturias, Instituto de Investigacion Sanitaria del Principado de Asturias, Oviedo, Spain; 16 Pneumology Department, Hospital Universitario Nuestra Señora de La Candelaria, Santa Cruz de Tenerife, Spain; 17 Pneumology Section, Hospital Sierrallana-TresMares, Cantabria, Spain; 18 Department of Respiratory Medicine, University Hospitals of Coventry and Warwickshire, Clifford Bridge Road, Coventry, UK; 19 Servicio de Neumología. Hospital Clínico Universitario San Cecilio. Departamento de Medicina, Facultad de Medicina, Universidad de Granada, Granada, Spain; 20 Clinic for Respiratory Diseases, University Hospital Center Zagreb, Zagreb, Croatia; 21 School of Medicine, University of Zagreb, Zagreb, Croatia; 22 Laboratory of Respiratory Diseases, Department of Chronic Disease, Metabolism and Ageing, Katholieke Universiteit (KU) Leuven, Leuven, Belgium; 23 Department of Respiratory Diseases, University Hospitals Leuven, Leuven, Belgium; 24 Servicio de Neumología, Hospital Clínico Universitario de Valencia. Instituto de Investigación INCLIVA, Valencia, Spain; 25 Department of Pulmonology, Leiden University Medical Center, Leiden, The Netherlands; 26 Division of Pulmonology, University Hospital Zurich, Zurich, Switzerland

**Correction to: Respiratory Research (2022) 23:352** 10.1186/s12931-022-02275-4

Following publication of the original article [1], the authors identified an error in Fig. 2 legends. The legend says KOC (%), but it should be KCO (%).

The correct figure (Fig. 2) is given in this correction.

**Fig. 2:**
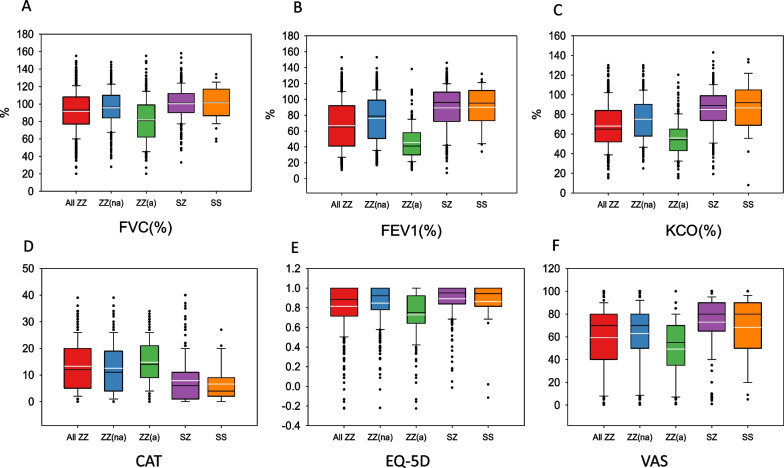
Distribution of values of lung function and quality of life according to the different genotypes. ZZ(na): non-augmented PI*ZZ; ZZ(a): Augmented PI*ZZ. In each box plot, the median value is indicated by the center horizontal black line, the 25th and 75th percentiles are indicated by the lower and upper box horizontal lines, and the mean value is indicated by the center horizontal white line. Whiskers above and below the box indicate the 90th and 10th percentiles. Circles on the high end indicate the outliers

The original article has been updated.

## References

[1] Miravitlles M, Turner AM, Torres-Duran M, Tanash H, Rodríguez-García C, López-Campos JL, Chlumsky J, Guimaraes C, Rodríguez-Hermosa JL, Corsico A, Martinez-González C, Hernández-Pérez JM, Bustamante A, Parr DG, Casas-Maldonado F, Hecimovic A, Janssens W, Lara B, Barrecheguren M, González C, Stolk J, Esquinas C, Clarenbach CF (2022). Clinical and functional characteristics of individuals with alpha-1 antitrypsin deficiency: EARCO international registry. Respir Res.

