# Supplementary material for: Clinical and functional characteristics of individuals with alpha-1 antitrypsin deficiency: EARCO international registry
Source: Respir Res. 2022 Dec 16;23:352. doi: 10.1186/s12931-022-02275-4 (PMC9756677; doi:10.1186/s12931-022-02275-4)
Supplement: Supplementary file 1 — Additional file 1: Table S1. Description of rare variants identified in EARCO. [file 12931_2022_2275_MOESM1_ESM.docx]

**Table S1**. Decsirption of rare variants identified in EARCO

| **Genotypes** | **Number** |
| --- | --- |
| Mmalton/Z | 10 |
| Mmalton/S | 9 |
| Q0/Z | 7 |
| S/I | 6 |
| Q0/Q0 | 4 |
| Z/F | 3 |
| Mlike/S | 3 |
| Plovel/S | 3 |
| Mheerlen/S | 2 |
| Plovel/Z | 2 |
| Q0/S | 2 |
| Mmalton/Mmalton | 2 |
| Mlike/Z | 2 |
| Mmalton/Mprocida | 1 |
| Mvalld’hebron/S | 1 |
| Mprocida/S | 1 |
| Mwurzburg/S | 1 |
| Mmattawa/S | 1 |
| Z/? | 1 |
| Mpalermo/Z | 1 |
| Mwurzburg/Z | 1 |
| Z/I | 1 |
| Msevilla/Z | 1 |
| Undetermined | 4 |
